# Chitosan–Clay Mineral Nanocomposites with Antibacterial Activity for Biomedical Application: Advantages and Future Perspectives

**DOI:** 10.3390/ijms251910377

**Published:** 2024-09-26

**Authors:** Danina Krajišnik, Snežana Uskoković-Marković, Aleksandra Daković

**Affiliations:** 1 Department of Pharmaceutical Technology and Cosmetology, Faculty of Pharmacy, University of Belgrade, 11221 Belgrade, Serbia; 2 Department of Analytical Chemistry, Faculty of Pharmacy, University of Belgrade, 11221 Belgrade, Serbia; snezana.uskokovic@pharmacy.bg.ac.rs; 3 Institute for Technology of Nuclear and Other Mineral Raw Materials (ITNMS), 11000 Belgrade, Serbia; a.dakovic@itnms.ac.rs

**Keywords:** chitosan, clay minerals, bionanocomposites, preparation, characterization, antibacterial activity, biomedical application

## Abstract

Polymers of natural origin, such as representatives of various polysaccharides (e.g., cellulose, dextran, hyaluronic acid, gellan gum, etc.), and their derivatives, have a long tradition in biomedical applications. Among them, the use of chitosan as a safe, biocompatible, and environmentally friendly heteropolysaccharide has been particularly intensively researched over the last two decades. The potential of using chitosan for medical purposes is reflected in its unique cationic nature, viscosity-increasing and gel-forming ability, non-toxicity in living cells, antimicrobial activity, mucoadhesiveness, biodegradability, as well as the possibility of chemical modification. The intuitive use of clay minerals in the treatment of superficial wounds has been known in traditional medicine for thousands of years. To improve efficacy and overcome the ubiquitous bacterial resistance, the beneficial properties of chitosan have been utilized for the preparation of chitosan–clay mineral bionanocomposites. The focus of this review is on composites containing chitosan with montmorillonite and halloysite as representatives of clay minerals. This review highlights the antibacterial efficacy of chitosan–clay mineral bionanocomposites in drug delivery and in the treatment of topical skin infections and wound healing. Finally, an overview of the preparation, characterization, and possible future perspectives related to the use of these advancing composites for biomedical applications is presented.

## 1. Introduction

According to the IUPAC definition, a composite material is a multi-component material comprising multiple, different (non-gaseous) phase domains in which at least one type of phase domain is a continuous phase [1]. Further, in the literature, composites are defined as “a material consisting of two or more different materials combined to create a superior and unique material” [2] or “composites or composite materials are engineered materials that consist of two or more constituent materials with wide discrepancies in their physical, chemical and mechanical properties” [3]. The use of composite materials has a very long tradition, and nowadays, they are used as construction materials in the automotive industry, aerospace, housing, food packaging, and in medical fields as biomaterials for various applications [3,4,5,6,7].

In general, the structure of a composite material consists of a matrix phase, which is usually continuous, and a dispersed phase, also known as the reinforcing phase. There are several classifications of composites based on the properties of the matrix and the reinforcing materials. The main types of matrices are polymers, metals, and ceramics. Depending on the type of reinforcement phase, there are the following common types of composites: (a) particulate composites, (b) fiber composites, and (c) lamellar composites. Various materials are also used as reinforcement, including glass, carbon, aramid, ceramics (e.g., silicon carbide or alumina), polymers, and metals. The composites can be categorized into natural and synthetic composites according to the nature of the constituents’ phases, while the composites can be referred to as nanocomposites, microcomposites, or macrocomposites, depending on their scale [2,8]. In nanocomposites, at least one constituent phase has one dimension or more in the nanoscale range, i.e., less than ~50–100 nm [8]. In addition, the term “hybrid” usually refers to composites whose components are at the nanometer or molecular level [9].

Chitosan is a natural polymer that possesses a wide range of commendable properties that have been exploited for biomedical research. It is known that this natural biomaterial is biocompatible, hydrophilic, and has broad antimicrobial properties [10]. Clay minerals have been used for a long time for various purposes. Recently, due to their high specific surface areas, ion-exchange capacities, layer charges, and rheological and mechanical properties, clays have been intensively studied in various pharmaceutical and biomedical fields as perspective excipients to improve the technological and biopharmaceutical aspects of medicinal products. Clays are also being investigated as drug-delivery systems as they can increase solubility and permeability, control and prolong drug release and efficacy, reduce side effects, and thus increase safety [11].

In the field of biomedical applications, polymer composites have numerous advantages, such as low cost due to the use of available natural and synthetic matrices as well as simple manufacturing techniques [12].] The combination of natural biopolymers with nanoscale fillers enables the production of bionanocomposites that exhibit improved mechanical strength, thermal stability, and biocompatibility, making them ideal for tissue engineering, drug delivery, biosensors, and diagnostic imaging [13,14,15].

In modern pharmacy and medicine, there is a need to find advanced therapeutic systems that are also more rational, efficient, and environmentally friendly. It is well known that nanosystems for drugs can have many advantages, such as improving the solubility profile, controlling and targeted drug delivery, achieving maximum pharmacological effect with minimal side effects, reducing the frequency of administration, and increasing metabolic/enzymatic stability or protecting and stabilizing drugs from uncontrolled degradation during storage and in vivo [16]. Nanostructured materials can be used for the delivery of antibiotics or have antimicrobial activity themselves [17]. Chitosan-based nanoparticles are particularly interesting due to the specific properties of this natural cationic polymer with numerous beneficial properties, such as biocompatibility, biodegradability, mucoadhesiveness, and antimicrobial properties [18,19,20]. In addition, the use of nanomaterials, i.e., nanomedicines, to combat the high rates of antimicrobial resistance is considered an advanced solution for the fields of medicine and public health [21,22].

This review summarizes the description and key properties of chitosan–clay bionanocomposites prepared with the most studied clays (montmorillonite and halloysite) and their recent biomedical applications, particularly in drug delivery and wound healing. The antibacterial activity of chitosan–clay bionanocomposites will also be discussed. In addition, the structure, physicochemical properties, and safety of chitosan and chitosan–clay bionanocomposites are illustrated for a better understanding.

## 2. Chitosan–Clay Nanocomposites

The term “biocomposites” or “green composites” is commonly used to refer to composite materials in which either the matrix or the reinforcement or both are made from renewable, biodegradable resources [23]. Bionanocomposites combining biopolymers with nanoscale reinforcements, typically in the form of nanoparticles, nanotubes, or nanofibers in the range of 1–100 nm, have received considerable attention due to their unique advantages such as excellent biodegradability, availability, cost-effectiveness, and environmental friendliness [14,24]. Bionanocomposites prepared from natural polymers (especially polysaccharides) and inorganic materials have attracted tremendous interest due to the hybrid properties of both components. Among them, bionanocomposites based on chitosan and inorganic clay particles (nanoparticles) have been intensively studied in the last two decades due to their excellent properties suitable for biomedical applications [23,25,26,27,28].

### 2.1. Chitosan

**Figure 1:**
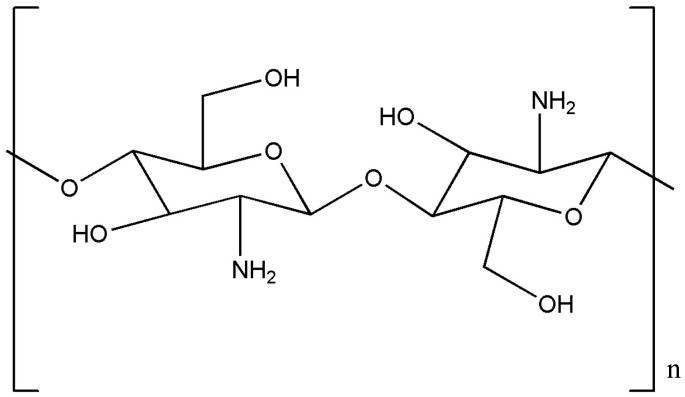
Chemical structure of chitosan.

Nowadays, it is imperative to apply eco-friendly ingredients, especially bio-waste-originated materials, for a wide range of applications that perfectly fit the polysaccharide chitosan. Chitosan is a nature-based material with an incredibly wide range of properties and intriguing applications [29]. Regarding structure, chitosan consists of randomly β-(1→4)-linked deacetylated and N-acetylated units of D-glucosamine (Figure 1).

Chitin, which is a structural element in the exoskeleton of crustaceans (preferably crab and shrimp), can be used as a starting material for alkaline deacetylation to produce chitosan. More recently, the rational process of obtaining chitosan is from the waste material during the cultivation of fungi because it is found in their cell walls. Due to its properties, such as biocompatibility and biodegradability, chitosan can be declared as a biomaterial and, therefore, has a harmless effect on humans, animals, and nature [30]. Synonyms of chitosan, such as deacetylchitin and poly-(D)glucosamine, refer to the structure and method of production [31]. Chitosan is a weak base due to the presence of amino groups in the glucosamine monomer units (Figure 1), and its pK_a_ value ranges from 6.4 to 6.7 depending on the ratio of glucosamine/N-acetyl glucosamine monomers [32]. Two hydroxyl groups in its monomer structure enable suitable chemical modifications [33]. Chitosan is insoluble in water and common organic solvents and soluble in dilute aqueous acidic solutions (e.g., acetic acid, formic acid, and nitric acid) [34].

The degree of deacetylation (DD) is an important characteristic of the final product that affects other properties. The usual range of the deacetylation degree of commercially available chitosan is 70–90%, while the molecular weight (MW) is about 50–2000 kDa [35]. Therefore, chitosan can be categorized into low- (<150 kDa), medium- (150–700 kDa), and high-molecular-weight (>700 kDa) groups according to its MW [36]. Perhaps the most attractive of these is low-molecular-weight (LMW) chitosan. The already confirmed antibacterial activity and biodegradability make LMW chitosan a good candidate for use in animal husbandry and agriculture [37].

Thus, because of the advantageous properties—good biocompatibility, biodegradability, nonallergenicity, film-forming capacity, and antioxidative and antibacterial activity—chitosan found great potential in biocompatible composites production, enabling a wide range of applications, such as food, cosmetics, water treatment, membranes, environmental protection, materials development, biomedicine, etc.

Although chitosan is commonly regarded as a biocompatible and safe biopolymer that is generally considered non-toxic and non-irritating [31], it has not yet been included in the FDA database of Generally Recognized As Safe (GRAS) food substances [38]. This could be due to its biological activity, structural versatility, and biodegradation variability, as well as distribution pathways, which largely depend on its structural properties, especially on MW and DD [30,39]. However, it is considered a pharmaceutical excipient and is included in both the Ph. Eur. and USP pharmacopeia monographs [31].

The biodegradability of chitosan is mainly due to its sensitivity to hydrolysis by the proteolytic enzyme lysozyme, which is present in all human tissues, and to lipase, an enzyme present in saliva and gastric and pancreatic juices. The products of enzymatic degradation are also non-toxic [40]. Chitosan binds effectively and non-selectively to mucosal surfaces in the biological environment. The cationic nature and electrostatic interactions are thought to be responsible for the occurrence of primary forces within mucoadhesion resulting from the reaction of positively charged amino groups of chitosan and negatively charged sialic acid residues in mucin, the basic glycoprotein of mucus. In addition, the hydrogen-bonding and hydrophobic interactions also play a role in the binding of chitosan molecules to mucosal surfaces [41]. Due to its positive charges in an acidic medium, chitosan can also interact with the negative charges of the cell membrane, leading to the reorganization and opening of the tight junction proteins, which explains the permeation-enhancing properties of chitosan [23].

Chitosan and its derivatives with controlled physicochemical properties have been shown to have numerous effects, such as antioxidant, antimicrobial, anti-inflammatory, antitumor, antidiabetic, wound-healing, and other positive influences on human and animal health [42]. For this reason, they have been used for years as food supplements in human and animal nutrition [37,43,44] or in products with hemostatic properties, such as chitosan-based hemostatic wound dressings [45,46]. Although the broad-spectrum antimicrobial activity of chitosan indicates great potential for this natural polymer, it is often associated with its physicochemical properties and depends on the type of microorganism [18].

Chitosan has antimicrobial activity against Gram-positive bacteria (such as *Bacillus cereus*, *Bacillus megaterium*, *Lactobacillus* spp., and *Staphylococcus aureus*); Gram-negative bacteria (such as *Escherichia coli*, *Enterobacter sakazakii*, *Pseudomonas aeruginosa*, *Pseudomonas fluorescens,* and *Salmonella typhimurium*); yeasts such as *Candida*, *Saccharomyces*, and *Rhodotorula;* and molds such as *Aspergillus*, *Penicillium*, and *Rhizopus* [42,47]. The mechanism of antimicrobial action of chitosan involves several processes, including electrostatic attraction, membrane permeabilization, and enzyme inhibition [48]. Physicochemical properties such as MW, DD, chitosan concentration, and pH of the medium also influence its antimicrobial activity in addition to the chitosan source [20]. In addition, biological conditions such as pH, temperature, salinity, and the presence of divalent cations should also be considered [46,47].

From a technological viewpoint, chitosan is an excellent film-forming material with selective gas permeability and good mechanical properties. This property is very important for application in, e.g., wound dressings, where one of the strategies to improve the mechanical properties of chitosan films is the incorporation of clays [33].

**Figure 2:**
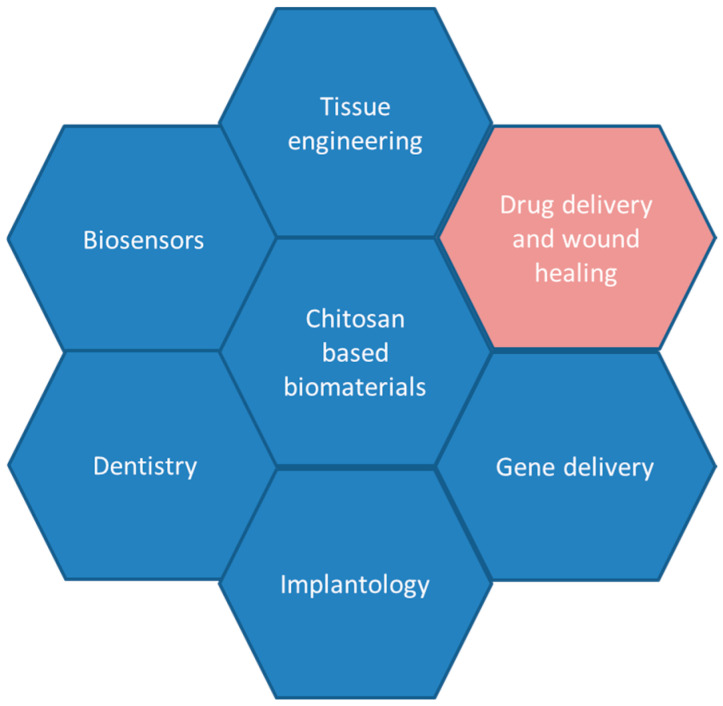
Schematic representation of the biomedical applications of chitosan-based bio-nanomaterials.

Bionanomaterials based on chitosan with improved properties of the starting material have found potential uses in biomedicine, pharmaceutical, food, and agro-industry, as well as environmental protection [49]. Biomedical applications of these materials exhibit a high potential in tissue engineering, drug delivery, gene delivery, wound healing, implantology, dentistry, and biosensors (Figure 2) [50,51,52,53].

Unfortunately, besides the already numbered advantages of chitosan, there are several limitations that prevent its bioactivity, such as low solubility in physiological environments and high viscosity even in dilute acidic solutions [54]. The creation of novel composites based on chitosan with the aim of enhancing its parent characteristics opens new pathways for additional applications [29]. The economic aspect due to low-cost ingredients and procedures is another valuable and promising point of view in this field.

### 2.2. Nanoclays

Clay minerals are naturally occurring, layered mineral materials that are low-cost and environmentally friendly and are known as one of the oldest materials used in traditional medicine. These materials possess specific physicochemical characteristics such as high surface reactivity (adsorption and cation-exchange capacity), colloidal and swelling properties, optimal rheological behavior, and high water dispersibility that make them suitable for various biomedical applications [55,56]. Most clay minerals are obtained by hydrothermal processing of alkaline volcanic ash and are mainly based on phyllosilicates [57]. Chemically, phyllosilicates (from the Greek “phyllon”, leaf, and from the Latin “*silic*”, flint) are hydrated aluminosilicate minerals consisting of aluminum and silicon oxides and contain many cations such as Mg^2+^, K^+^, Ca^2+^, Na^+^, and Fe^3+^ [58]. Structurally, phyllosilicates consist of continuous, stacked, tetrahedral SiO_4_ and octahedral AlO_6_ sheets [59]. The thickness of each layer is approximately 1 nm, and the lateral dimensions of these layers vary between 100 and 500 nm, while the space between the layers can be empty or contain hydrated alkali and alkaline earth cations forming the “structural units”. The primary particles of clays are formed by overlapping 5–10 parallel layers and producing so-called primary particles. Random grouping of the primary particles results in an aggregate whose dimension can be up to 10 μm [11,60,61,62].

Clays are often classified based on their specific structures and the different ratios of the sheets (layers), cation-exchange capacity (CEC), interlayer space (d-space), morphology, swelling capacity, or surface charge [55,57].

The basic structural units of phyllosilicates are aluminosilicate layers that are formed by the combination of the main components—tetrahedral and octahedral sheets—which are bound together by sharing oxygen atoms. Based on the way that the tetrahedral and octahedral sheets are packed into layers, clay minerals can be classified as (a) a 1:1 type of clay mineral in which one octahedral layer is linked to a tetrahedral one—kaolinite, halloysite, and rectorite; (b) a 2:1 type of clay mineral, in which two tetrahedral sheets are on either side of an octahedral one—pyrophyllite, smectite (montmorillonite, hectorite, and saponite), vermiculite, mica, and illite; and (c) a 2:1:1 type of clay mineral, with a positively charged brucite sheet sandwiched between layers that restrict swelling (chlorite) [63,64].

Based on their charge, clay minerals can be generally divided into groups: cationic clays, which possess a negative charge and are widespread in nature (e.g., smectite), and anionic clay minerals, which possess a positive charge (e.g., synthetic layered double hydroxides (LDH)) [55]. Due to the high CEC and the large total surface, cationic clays can be modified/functionalized with various organic substances such as drugs or polymers, which can be localized on the surface, at the edges, or in the interlayer spaces of the clay particles [55,65,66].

Clay minerals have been referred to as “nanoclays” due to their layers that are on the nanoscale scale [67], i.e., with at least one dimension in the order of 1–100 nm [56,61]. According to ISO/TS 21236-1:2019 [68]: “clay nanomaterial is material composed predominately of clay with any external dimension in the nanoscale or having an internal structure or surface structure in the nanoscale”. The dimensions of these materials also fulfill the definition of nanoscale materials according to EU [69] and US regulations [70,71].

Some representatives of the clays (e.g., kaolinite, talc, smectite, and palygorskite) have been used for a long time in pharmaceutical products both as active ingredients and as excipients (auxiliary substances). Bentonite, the raw material (rock) mainly consisting of montmorillonite mineral, as well as talc and kaolin—the rock rich in kaolinite—are included as pharmaceutical excipients in the monographs of the pharmacopeias (Ph. Eur., USP) and must therefore be fully characterized and have satisfactory pharmacopeial quality [31,72,73]. Considering the application of clays in other biomedical fields, such as drug delivery, wound healing, biosensors, tissue engineering, etc., many studies are focused on montmorillonite and halloysite [56,61].

**Figure 3:**
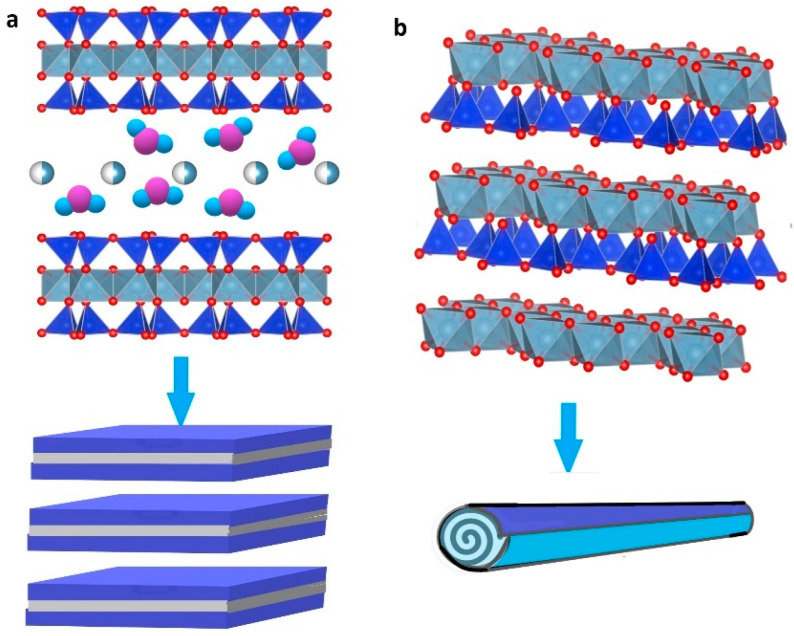
Schematic representation of montmorillonite (**a**) and halloysite (**b**).

Montmorillonite (Na,Ca)_0.33_(Al,Mg)_2_(Si_4_O_10_)(OH)_2_·*n*H_2_O) [63] is a representative of the smectite group of clays (2:1 phyllosilicates) with a distinctive layered platelet morphology (Figure 3a). In montmorillonite, some trivalent aluminum ions are often replaced by divalent magnesium ions in some sites of the octahedral layer, and some silicon ions in the tetrahedral layer may be replaced by aluminum ions, giving rise to a negative charge of the unit cell of montmorillonite mineral. This negative charge is typically balanced by the exchangeable hydrated alkali or alkaline earth cations, and the sum of these exchangeable cations is expressed as a CEC of montmorillonite mineral. Depending on the type of exchangeable cations, the interlayer space of montmorillonite varies between 12 Å (characteristic for montmorillonite in which sodium is the main exchangeable cation hydrated with one water layer) and 15 Å (corresponding to a two-water-layer hydration state of alkaline-earth-smectite) [74]. The thickness of the montmorillonite silicate layer is ~1 nm, while the sidewall (thickness) of the clay mineral can vary from 30 nm to several micrometers or even more. This structure allows the penetration of water or external molecules (e.g., polymers) between the layers, which makes montmorillonite a good candidate for the preparation of polymer bionanocomposites via partial or complete intercalation or the exfoliation of clay [23,56,57].

Halloysite (with the general stoichiometry Al_2_Si_2_O_5_(OH)_4_·nH_2_O, where *n* = 4 for 1.0 nm wall-packing spacing and *n* = 2 for 0.72 nm (dried sample)) [63] belongs to a 1:1 layer type of clay minerals as kaolinite, with a single sheet of water molecules between two layers with interlayer spacing of 10.1 Ǻ, which make it different from kaolinite (interlayer spacing of 7 Ǻ). In contrast to the platy form of kaolinite, halloysite possesses a hollow tubular geometry with a length of 200 to 1500 nm, an outer diameter of ~ 50 nm, and an inner diameter of 10 to 15 nm (Figure 3b) [75,76]. The good biocompatibility, in combination with its nanotubular geometry, i.e., a negatively charged outer surface and a positively charged inner surface, makes this material particularly interesting for the preparation of polymer bionanocomposites. In addition, halloysite is more hydrophilic than other clay minerals and does not require any exfoliation during the composite preparation due to its hollow shape and lack of stacked layers [57,77,78].

Based on published data [79,80,81], natural clay minerals revealed no or only minor antibacterial effects. However, if organic (surfactants and biopolymers) or inorganic (copper, zinc, and silver) antibacterial agents are present in these minerals, they may have two different properties: (1) to adsorb different molecules and (2) to kill bacteria [82,83].

### 2.3. Preparation and Characterization of Chitosan–Clay Nanocomposites

Several methods have been developed for the preparation of polymer/clay bionanocomposites. Basically, during preparation, the polymer chains are placed between the clay mineral layers or polymerized from the desired monomers/precursors between the clay layers. Some of the first methods used for their preparation were solution-induced intercalation/solvent intercalation, in situ polymerization/intercalation, and melt processing/melt intercalation [4,84]. According to the literature, the successful formation of a polymer–clay bionanocomposite depends on the ability to modify the chemical structure of the aluminosilicate via ion-exchange reactions with organic or inorganic cations to produce a polymer-compatible nanocomponent and the ability of the aluminosilicate particles to disperse in the polymer, resulting in an exfoliated bionanocomposite [62].

**Figure 4:**
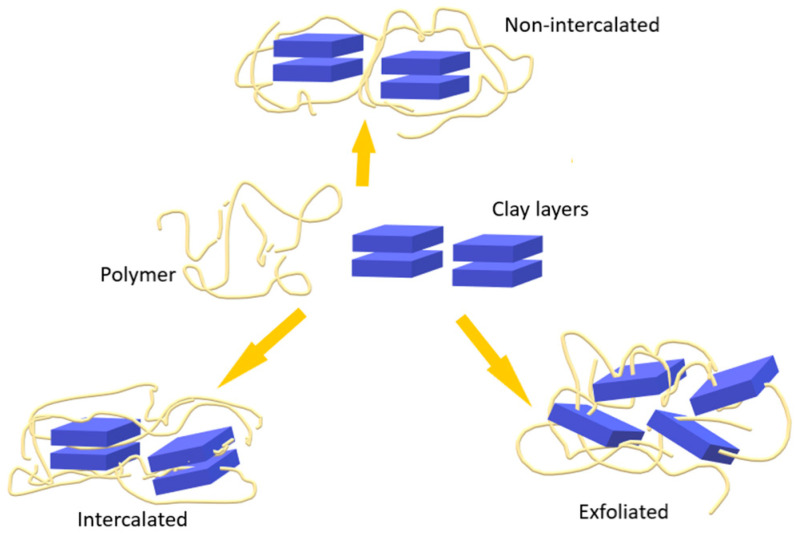
Polymer–clay composite structures formed by the interaction between polymers and lamellar clays.

Based on the preparation method and the materials used, the morphology of the dispersion (microstructure) can be classified as (a) non-intercalated (microcomposite or tactoid structure), (b) intercalated (and/or flocculated), and (c) exfoliated (or delaminated) (Figure 4) [23,57,85].

In solution-induced intercalation, the inorganic particles are dispersed in a polymer solution. This type of composite is easier to produce with polymers and lamellar clay minerals, both having hydrophilic properties. In this case, the layered mineral must be swollen in a polymer solution to allow the diffusion of polymer molecules into interlayer spaces [23,39,72,86,87]. This method of preparation is suitable for water-soluble polymers, and since water is used as a solvent, it is considered a low health and safety risk for commercial production [4]. In situ polymerization is another method of intercalation in a liquid medium whose importance is related to its greater ability to produce uniformly dispersed nanocomposites in the monomer solution. Polymer precursors are introduced between swelling clay layers, which are subsequently expanded and dispersed in the matrix, with polymerization triggered by an external stimulus (thermal, chemical, or radiation) [4,72]. The melt processing is considered sustainable for environmentally friendly production, as the use of organic solvents is avoided [88]. In this process, melting leads to the intercalation of clays and polymers; therefore, it is dependent on the polymer-processing conditions in the molten state, e.g., extrusion [4,72]. Polymer/clay bionanocomposites for biomedical applications can be processed into various morphologies, e.g., 2D or 3D thin films, scaffolds, hydrogels, fibers, and bioinks for 3D printing [57,89].

Various techniques can be used to characterize chitosan–clay bionanocomposites. X-ray diffraction (XRD) is used as a versatile and non-destructive method to determine crystal structures, evaluate polymorphic and solvate structures, determine the degree of crystallinity, and study phase transitions [27]. Along with scanning electron microscopy (SEM) and transmission electron microscopy (TEM), these techniques are powerful tools for analyzing the morphology and microstructure of various nanocomposites [86,90,91,92]. FTIR spectroscopy and zeta potential measurements are often used to study the interactions between chitosan and nanoclays [93,94,95,96]. Atomic force microscopy (AFM) is suitable for studying the nanotopography of chitosan–nanoclay composite films [97,98]. Thermal analysis, differential scanning calorimetry (DSC) and thermogravimetric analysis (TGA), represent the first-order analytical technique for accurate physicochemical solid-state characterization and thermal properties of clay minerals and polymers as well as their bionanocomposites [86,92,94,99].

Investigation of functionality-related properties of chitosan–clay bionanocomposites is very important for their practical use. The nanocomposite films and hydrogels can be characterized by examining the degree of swelling, porosity analysis, folding endurance, thickness, and mass measurement or water vapor permeability [100,101], while their mechanical properties can be determined by various tensile strength tests [97,98,102].

Chitosan is a biopolymer with known antibacterial activity [18,103], and therefore, the investigation of the antibacterial activity of chitosan–clay nanocomposites is usually carried out using reference bacterial and fungal strains [82,98,99]. For example, Cankaya and Sahin [99] prepared biopolymer/clay nanocomposites via modification of Na^+^montmorillonite (C Na), nanoclay 1–135 (C 10A), and nanoclay 1–140 (C 15A) with chitosan (average MW of 310–375 kDa) using the solution induced intercalation method with various amounts of the three clays. They tested the antimicrobial properties of nanocomposites toward *S. aureus* and *E. coli* and reported the highest antimicrobial activity for the chitosan/C 10A biocomposite. Lertsutthiwong et al. [95] modified sodium montmorillonite with chitosans of different average MW: 71, 220, and 583 kDa and DD of 85–90%. The highest amount of intercalated chitosan was achieved by the addition of chitosan with a MW of 71 kDa and DD of 80% at a fixed chitosan/montmorillonite mass ratio of 2:1. This composite showed good adsorption of the cationic dyes and also inhibited *E. coli* by almost 100%.

Several techniques, such as the dialysis bag technique, the paddle method, or the Franz diffusion cell, have been used for in vitro drug release from bionanocomposites containing active substances [27,94,104,105,106,107].

The evaluation of cell toxicity, in vivo wound healing, antioxidant activity, and histological analysis are some of the tests performed to investigate the safety and efficacy of chitosan–clay bionanocomposites of different morphologies such as hydrogel composites, nanocomposite films, and nanocomposite powders [100,101,102,108,109,110,111].

An overview of chitosan–clay bionanocomposites with antibacterial activity for various biomedical applications and methods of their characterization are given in Table 1.

## 3. Chitosan–Clay Nanocomposites for Biomedical Applications

**Figure 5:**
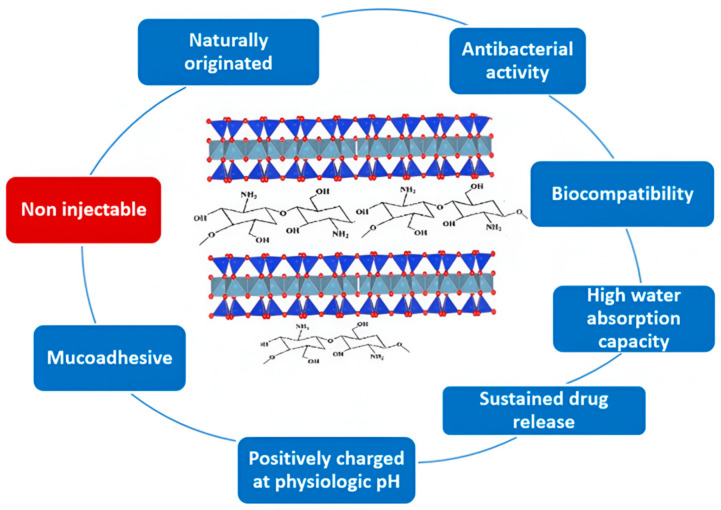
Key features of chitosan–clay nanocomposites relevant to their biomedical applications.

Polymer–clay bionanocomposites have been extensively studied for various biomedical applications such as tissue engineering, drug delivery and wound healing, scaffold and bone cement fabrication, cancer therapy, and enzyme immobilization [56,57,112]. Chitosan–clay bionanocomposites are being extensively studied for drug delivery and wound healing due to their unique properties (Figure 5) that combine the properties of nanoclays (specific morphology, ion exchange and swelling capacity, mechanical properties, etc.) and properties of biopolymer–chitosan (biocompatibility, film-forming ability, and antibacterial properties) [29].

### 3.1. Drug Delivery

Novel clay–drug hybrid materials have been investigated as drug carriers due to their advantageous properties, such as biocompatibility, increased drug solubility and adsorption, prolonged drug release, mucoadhesion, targeting, etc. [113]. As the literature already underlines, chitosan’s cationic nature is a must-have characteristic for making an aluminosilicate composite because of the ion exchange interaction on which the composite forming is based [39]. Further, a high surface-to-volume ratio and remarkable chemical and mechanical stability of aluminosilicates, in combination with chitosan properties, may result in potent drug-delivery composite materials.

Among the various routes of administration, local drug delivery is particularly interesting as it allows the drug to be administered directly to the target organ while maintaining effective concentration and minimizing systemic side effects [114]. Dosage forms/carriers for local administration based on clay minerals investigated to date include buccal films and tablets, bone cement, preparations for periodontal treatment, films or implants for ocular drug delivery, and skin/topical applications [33,56,113].

Antimicrobial agents for topical application can be classified as antiseptics; antibiotics; and plants or plant-derived forms, such as essential oils; and oleoresins [115]. Antibiotics are defined as natural (produced by microorganisms) or synthetic compounds that, in a low concentration, exhibit microbial static or microbicide effects. They are more or less nontoxic and, nowadays, unfortunately, more and more susceptible to bacterial resistance. In order to minimize the development of resistant organisms, it is advisable to limit the choice of topically applied antibacterial agents to those that are not used systemically. Unfortunately, some of them, e.g., neomycin, are sensitizing, and there is cross-sensitivity with other aminoglycoside antibiotics, such as gentamicin. When large areas of skin are treated, ototoxicity may also occur with aminoglycoside antibiotics, particularly in children, the elderly, and people with renal impairment [116]. The most frequently used antibiotics for topical application, as well as their basic characteristics, are summarized in Table 2.

Two important inconveniences exist regarding the topical application of antibiotics. The first is the development of bacterial resistance against used antibiotics [118]. One way to avoid this increasing problem could be to employ nanoparticles [21,119]. The use of antibiotic-loaded nanoparticles is considered a valid strategy as it offers numerous advantages over conventional formulations, including improved stability, controlled release of antibiotics, targeted action, and increased bioavailability [120]. In addition, nanoparticles can attack biofilms and treat resistant pathogens due to their special size and physical properties [22]. The mucoadhesive character of chitosan nanoparticles contributes to their prolonged residence time on the biomembranes and therefore enables their application by various routes (e.g., oral, nasal, pulmonary, vaginal, ocular, buccal, or skin) [120,121]. Another significant advantage of these bionanocarriers is the inherent antimicrobial activity of chitosan. Contact allergy to antibacterial topical application is another possible issue that must be considered and avoided. Both possible disadvantages could be avoided by creating chitosan–clay bionanocomposites loaded with antibiotics. Besides more comfort for patients, it is expected that the necessity of lowering the concentrations, with controlled release over time [122], will be necessary to achieve the desired result.

Montmorillonite and halloysite functionalized with chitosan are the most cited aluminosilicates in the literature, with drug-delivery purposes and wound-healing, as well. Antibiotics, such as silver-sulfadiazine [33] and tetracycline [107], are examples of loaded drugs onto chitosan-aluminosilicates. Čalija et al. [107] investigated the functionality of tetracycline-loaded chitosan-halloysite nanocomposite films, with a focus on the evaluation of the influence of chitosan MW on film applicability for sustained local tetracycline delivery. The films were prepared with halloysite and low-, medium-, or high-MW chitosan. They found that the nanocomposite chitosan-halloysite films released tetracycline in such a way that the slowest release was achieved from the films consisting of LMW chitosan. These findings provide the possibility of moderating the final drug release profile.

Ghazaie et al. [82] intercalated ciprofloxacin in different amounts (10–40%) into the Na^+^-montmorillonite at two different pH values (5 and 7) and obtained materials subsequently coated with N,N,N-triethyl chitosan. They compared the release of the drug from composites with and without the addition of chitosan and found that composites with chitosan possessed efficiency in drug adsorption and its controlled release in comparison with composites without coating with chitosan. The authors reported on the antibacterial activity of two composites containing ciprofloxacin (in amounts of 10 and 20%), montmorillonite, and chitosan against *S. aureus* and concluded that a composite with a lower amount of the drug showed significant antibacterial activity. Ambrogi et al. [104] prepared and characterized chitosan/montmorillonite composite films containing chlorhexidine—CLX (a good antimicrobial agent)—in order to achieve a prolonged drug release. They reported that the use of composite films of montmorillonite with chitosan and a loaded drug may be of interest for achieving a localized and prolonged release of chlorhexidine.

**Figure 6:**
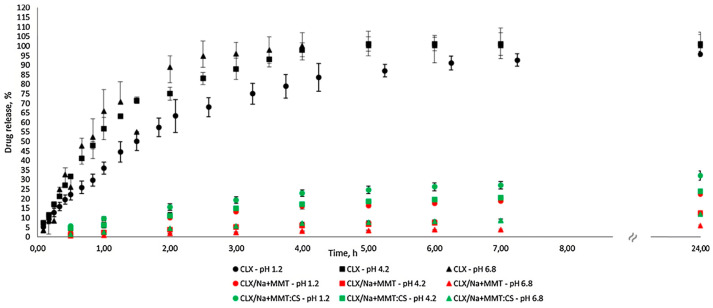
Release profiles of CLX formulations in different pH media (reprinted from Onnainty, R., Onida, B., Páez, P., Longhi, M., Barresi, A., & Granero, G. (2016). Targeted chitosan-based bionanocomposites for controlled oral mucosal delivery of chlorhexidine. *International Journal of Pharmaceutics*, 509(1–2), 408–418 [90], with permission from Elsevier).

Onnainty et al. [90] studied CLX release from a composite of montmorillonite (MMT) and chitosan (CS) obtained using the ion-exchange method. The release of pure CLX and CLX nanocomposites carriers with Na^+^MMT, with or without CS, was studied at three different pH values (1.2, 4.2, and 6.8) to mimic the pathophysiological conditions of the oral cavity and exploit the CLX nanocomposites’ usage as pH-responsive drug-delivery systems for treatment of bacterial infections. A long-term (during 24 h) sustained release of the drug from the obtained carrier without initial burst release was achieved (Figure 6). It was concluded that more controlled release profiles of CLX from the nanocomposite systems compared to the pure CLX offer the possibility of drug release carriers for applications where relatively slower profiles are desirable. The composite of montmorillonite with chitosan and loaded CLX also exhibited good mucoadhesion properties (evaluated by SEM), maintaining the drug’s antimicrobial properties.

Salcedo et al. [110] developed nanocomposites based on chitosan and montmorillonite using solid–liquid interaction as a carrier for the improvement in the oral bioavailability of oxytetracycline. For evaluation of in vitro cytotoxicity and drug permeation, Caco-2 cell cultures were used. The results confirmed the uptake of the nanocomposite into the cells, enhancing drug penetration and being biocompatible with Caco-2 cells. Luo and Mills [102] studied the effects of increased chitosan and halloysite nanotubes (HNTs) concentrations on the mechanical properties of chitosan/HNTs hydrogels, with and without the addition of gentamicin. They reported that the addition of HNTs to chitosan hydrogels improved the gels’ mechanical properties and that chitosan/HNTs composites containing gentamicin enabled prolonged gentamicin release (up to 104 h) and were effective in reducing bacterial growth (*E. coli* and *S. aureus*). Chlorhexidine salts can be included in the composite structure as an antiseptic active substance and are also used to treat skin cancer [123]. The combination of chitosan, aluminum silicate, and CLX in dentistry has already been confirmed as potent and multifunctional [10]. Another important possibility for the local application of composite carrier systems is the treatment of infections of the musculoskeletal system, such as osteomyelitis [124,125,126]. Chitosan sponges [127,128] and films [129] have been shown to be effective in the local administration of antibiotics. The results obtained so far, which include inorganic nanoparticulate carriers for the local delivery of antibiotics [130,131,132,133], encourage further investigations in this field and the application of chitosan–clay bionanocomposites.

### 3.2. Wound Healing

The skin is considered the largest organ in the body, estimated to account for about 15% of the total body weight. In addition to covering an entire external surface, the skin plays a huge role in temperature regulation and tactile sensation and is composed of three layers—the epidermis, dermis, and hypodermis. Among numerous functions is protection against ultraviolet light, trauma, pathogens, microorganisms, and toxins. The integrity and health of the skin are imperative because any type of skin damage poses a threat to the well-being of the body, and every wound must be restored as soon as possible [134].

Although they look like synonyms, the terms “wound healing” and “skin regeneration” differ [135]. The wound-healing process can be explained as a complex cascade route, ending with the stage during which an already formed matrix is remodeled into functional skin or semi/non-functional scar tissue [136].

In brief, wound healing includes four important phases: (a) hemostat, (b) inflammatory, (c) cell proliferation, and (d) remodeling [137]. Due to the complex mechanism, wound-healing treatment can emphasize many kinds of antiseptics and antibiotics, a wide selection of vitamins or supplements, and dressings, as well [135]. The promising role of a bioengineered matrix for wound healing focuses on a non-immunogenic, biocompatible, bio-resorbable, porous structure with sufficient mechanical strength. Natural biopolymers, including chitosan, have been successfully tested for this purpose [138].

Biomaterials with mechanical, biological, and chemical properties appropriate for wound-healing processes are the material of choice compared to synthetic ones [139]. Biopolymers are especially welcomed in this field because of their affordable resources, nontoxicity, and eco-friendly nature. Chitosan, a polysaccharide biopolymer, fulfills the most necessary characteristics to be the focus of research attention as a potent wound-healing material [140]. Chitosan modifications, such as photosensitizers, dendrimers, graft copolymerization, quaternization, carboxyalkyl chitosan, acyl chitosan, phosphorylated chitosan, sulfation, and thiolation, result in very wide possibilities in biomedical applications, with special potential in wound dressing and healing. In summary, some of the confirmed actions are wound dressing as a gauze; sustained drug delivery; antibacterial effects; antioxidant; excellent hemostatic, angiogenic, and anti-thrombotic properties; metal ion adsorption; controlled and targeted release of drugs; tissue engineering; and wound dressings [140].

In general, chitosan-based materials for wound healing can be applied as sponges, hydrogels, membranes, gels, and nanocomposites. An ideal wound dressing material is moisture-based, able to remove excess exudate, efficient in infection prevention, less adhesive, and, not less importantly, easy to remove and affordable [141]. Diabetic wounds are examples of non-healing wounds, where the healing process is blocked at an early stage and actually easily becomes chronic [142].

Examples of bionanocomposites of montmorillonite or halloysite with chitosan for wound healing are given below. In the study of Sandri et al. [111], nanocomposites were prepared with different amounts of montmorillonite (100–2000 mg) and 40 mL of a 1% (*w*/*w*) chitosan glutamate aqueous solution and subsequently loaded with silver sulfadiazine (AgSD) with the aim of preventing delay in wound healing. Antimicrobial properties of nanocomposite containing 100 mg of montmorillonite and chitosan loaded with AgSD were evaluated against *S. aureus*, *S. pyogenes*, *E. coli*, and *P. aeruginosa*. They reported that nanocomposite with AgSD showed good in vitro biocompatibility and gap-closure properties (fibroblasts). The material also maintained AgSD antimicrobial properties, especially against *P. aeruginosa*, which often complicates skin lesions. Moghadas et al. [98] prepared nanohybrid films with montmorillonite and chitosan sulfate chains using the solvent-casting method. The measured moisture vapor transmission rate of films indicated that films are good candidates as wound-dressing systems, and they showed significant antibacterial activity against *E. coli*.

**Figure 7:**
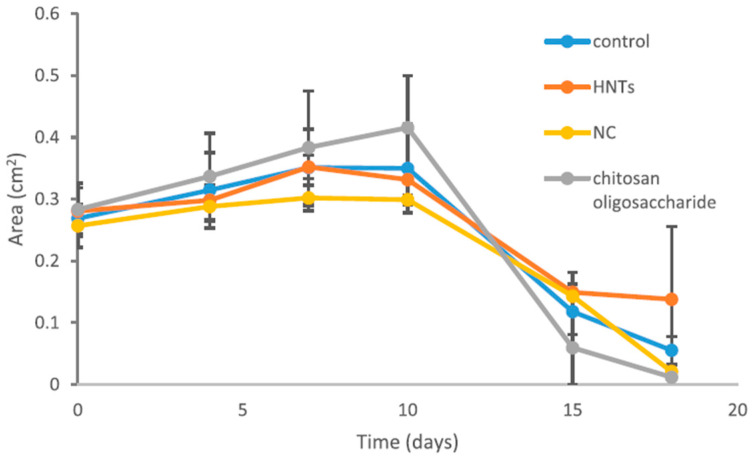
In vivo lesion reduction vs. time profile evaluated for the following samples: NC—0.05 chitosan oligosaccharide/HTNs nanocomposite (HNT concentration of 300 μg/mL and chitosan oligosaccharide concentration of 4 μg/mL); HNTs (concentration of 300 μg/mL); chitosan oligosaccharide (concentration of 4 μg/mL); saline solution—negative control (mean values ± sd; *n* = 8) (reprinted from Sandri, G., Aguzzi, C., Rossi, S., Bonferoni, M. C., Bruni, G., Boselli, C., Cornaglia, A. I., Riva, F., Viseras, C., Caramella, C., & Ferrari, F. (2017). Halloysite and chitosan oligosaccharide nanocomposite for wound healing. Acta Biomaterialia, 57, 216–224 [92], with permission from Elsevier).

In another study, the intercalation solution technique was used for the preparation of biopolymer chitosan/montmorillonite nanocomposites loaded with silver sulfadiazine for wound-healing purposes. The authors showed that silver sulfadiazine was effectively loaded in the structure of the three-dimensional nanocomposite consisting of the chitosan chains adsorbed within the clay mineral interlayers [86]. Devi and Dutta [100] tested chitosan-bentonite nanocomposite films obtained using the solvent casting method as a material for wound healing. They reported a good antibacterial activity of films against Gram-positive (*B. subtilis*) and Gram-negative (*E. coli*) bacteria. Salcedo et al. [109] prepared a nanocomposite of montmorillonite and chitosan that possess mucoadhesive properties in combination with lower hydration properties in an acidic environment compared with pure chitosan. Chitosan and the prepared nanocomposite exhibited good biocompatibility on Caco-2 cells, also showing a progressive reduction in the wound area. The results indicated an effective stimulation of Caco-2 proliferation by using both chitosan and a nanocomposite. Sandri et al. [92] investigated HNTs and chitosan oligosaccharide nanocomposite produced by spontaneous ionic interaction as a material for wound healing. They reported that HTNs and the nanocomposite (NC) showed good in vitro biocompatibility with normal human dermal fibroblasts and enhanced in vitro fibroblast motility, promoting proliferation and migration. The healing capacity of NC, as well as HNTs and chitosan oligosaccharide, was tested in a murine (rat) model. The NC showed better skin re-epithelization and reorganization compared to HNTs and chitosan oligosaccharide alone. In vivo lesion reduction vs. time (0, 3, 7, 10, 14 and 18 days) profiles are shown in Figure 7.

As can be seen, all samples led to a significant reduction in the width of the lesion area. The NC sample nanocomposite exhibited the smallest lesion area profile, which was almost flat until up to 10 days, while HNT, chitosan oligosaccharide, and saline had almost overlapping profiles, with a slight increase in lesion area between 7 and 10 days of treatment. Kelly et al. [106] developed a novel drug-delivery system for the treatment of periodontitis by loading tetracycline into halloysite and subsequently coating the obtained material with chitosan. The prepared formulation was preliminary tested under in vivo conditions in dogs by using a wound pocket creation model to determine levels of drug release, antimicrobial activity, and retentive ability. They reported that the novel drug system showed good retention in the wound pocket, effective drug amounts released locally, and good antibacterial activity over a 6-week period.

## 4. Challenges and Future Perspectives

The constant and growing need to find advanced therapeutic systems that are more rational, efficient, and environmentally friendly exists in modern pharmacy and medicine.

The advantages of the novel drug-delivery systems, such as improvement in the solubility profile, controlled and targeted drug delivery, achievement of the maximum pharmacological effect with minimal side effects, reduction in the frequency of administration, increase in the metabolic/enzymatic stability, and protection and stabilization of the drugs from uncontrolled degradation during storage and in vivo are well known [16]. Nanostructured materials can be used for the delivery of antibiotics and wound healing or have antimicrobial activity themselves [17].

Chitosan–clay bionanocomposites consisting of chitosan and natural (nano)clays have special characteristics due to their unique morphology, availability, tailored composition, improved mechanical and physical strength, chemical stability, biodegradability, low cost, barrier properties, and cell compatibility [89]. However, since chitosan–clay bionanocomposites belong to a category of nanocarriers, their appropriate characterization is crucial to control their desired in vitro and in vivo behavior [143]. For the potential practical application of any of these bionanomaterials, the first step is to establish appropriate guidelines and protocols for their preparation and characterization, which should be reproducible, environmentally friendly, and commercially acceptable without harming the active ingredient. In addition to the physicochemical characterization [144], the evaluation of the biological efficacy and technological properties to verify the safety of these functional materials requires carrying out appropriate toxicological studies. Regarding the safety of clays and bionanocomposites derived from them, the literature indicates that it is difficult to draw a definitive conclusion, and it is therefore recommended to proceed on a case-by-case basis to avoid possible risks to humans and the environment. Indeed, different clays have their own toxicological profiles that could be altered by their modifications, which are necessary for novel applications [62]. Based on the numerous results published to date, clay nanoparticles are generally significantly less toxic than other nanomaterials that play the same role in the production of bionanocomposites [145]. However, for ongoing and future research in this field, defined protocols are needed to compare the data obtained under in vitro and in vivo conditions for chitosan–clay bionanocomposites to evaluate their metabolism and mode of action [17], which is very important for their safety in biomedical applications.

The development of bionanomaterial-based therapeutics that could overcome the current pathways of acquired drug resistance is a hopeful strategy for the treatment of difficult-to-treat bacterial infections [22]. It is expected that research into chitosan–clay bionanocarriers for various application sites will enable effective clinical use with numerous antibiotics in therapy. In addition, many examples of chitosan–clay bionanocomposites have been developed for biomedical applications, e.g., implantation or wound healing, so the production of sterile products packaged in suitable containers is another important research topic. In summary, chitosan–clay bionanocomposites can only make their maximum contribution to modern therapy in the future if methods for their preparation and characterization are developed simultaneously, which would increase the potential of their biomedical application

## 5. Conclusions

Chitosan–clay bionanocomposites, composed of chitosan as a representative of natural polymers and natural (nano)clays, are particularly interesting materials for drug delivery and wound healing due to their specific properties, which combine the properties of nanoclays (specific morphology, ion exchange, and swelling capacity, mechanical properties, etc.) and the properties of biopolymer chitosan (biocompatibility, film-forming capacity, and antibacterial properties). In general, they are recognized as safe materials in various areas of life. In addition, the use of these nanomaterials in biomedicine to combat the high rates of antimicrobial resistance is seen as an advanced solution for the medical and public health fields. It is expected that further multidisciplinary investigations of chitosan–clay bionanocomposites will expand their biomedical applications, especially in drug delivery and wound healing, in the future.

## Figures and Tables

**Table 1:** An overview of literature data on chitosan–nanoclay composites with antibacterial activity for biomedical applications.

Constituents	Preparation Technique/ Final Carrier Morphology/Type	Main Features	Methods of Characterization	Reference
MMT (CEC: 71 meq/100 g)/CS (MW: 71, 220 and 583 kDa; DD: 85–90%) API:/	Ion-exchange reaction/ composite powder	Antibacterial activity	● FTIR, XRD, TGA, Zeta potential measurement, textural properties (Brunauer, Emmet, Teller method—BET) ♦ Antibacterial properties (*E. coli—*a model pathogenic bacteria) ◊ Dye adsorption	[95]
MMT (CEC: n/a)/CS (MW: n/a; DD: 98%) API:/	Solution intercalation/ composite powder	Mucoadhesivity Wound-healing properties	◊ Water uptake, mucoadhesion ♦ Cell viability, wound-healing	[109]
MMT (natural and modified with a quaternary ammonium salt, CEC: n/a)/CS (MW: 310–375 kDa; DD: ≥75%) API:/	Solution-blending method/composite nanoparticles	Improved heat resistance of bionanocomposites Swelling proportional to the amount of chitosan in the bionanocomposites	• FTIR, XRD, SEM, DSC/TGA ♦ Antibacterial activity (the reference bacterial strains: *S. aureus*, *E. coli*) ◊ Swelling study	[99]
MMT (CEC: n/a)/CS (MW: 81 kDa; DD: 85%) API:/	Solvent casting/nanohybrid films based on CS and biofunctionalized MMT with CS-sulfate chains (SMMT)	Improved physicochemical properties compared to plain MMT/CS nanocomposite films Antibacterial activity against *S. aureus* and *E. coli* Possible application in wound dressing for burns and chronic and diabetic wound infections with low to moderate exudate.	• FTIR, XRD, dynamic mechanical thermal analysis, thermal analysis (DSC and TGA) ♦ Antibacterial activity (the reference bacterial strains: *S. aureus*, *E. coli*), cytotoxicity (MTT test) ◊ Tensile tests, water uptake, moisture vapor transmission rate, wettability and surface tension, surface morphology analysis, and surface modulus pattern	[98]
BNT (CEC: n/a)/CS (MW: 100 kDa; DD: 79%) API:/	Solvent casting/ nanocomposite films	CS-BNT films exhibited good antibacterial activity Hemocompatibility Suitable for wound-care products	● FTIR, SEM ♦ Antibacterial activity (the reference bacterial strains: *B. subtilis*, *E. coli*), in vitro nonenzymatic hydrolytic degradation, hemocompatibility test ◊ Folding endurance, thickness and mass measurement, water-absorption capacity, water vapour transmission rate, dressing pH, porosity measurement	[100]
MMT (CEC: 80.64 meq/100 g)/CS (MW: 251 kDa; DD: 98%) API: silver sulfadiazine	Solution intercalation/ composite powder	Bacteriostatic and bactericidal properties, suitable for skin lesions	• Chitosan assay, silver sulfadiazine assay ♦ Biocompatibility and proliferation tests (normal human dermal fibroblasts), cell motility assay for wound healing, antibacterial activity (the reference bacterial strains: *S. aureus*, *S. pyogenes*, *E. coli*, and *P. aeruginosa*)	[111]
MMT (CEC: 80.64 meq/100 g)/CS (MW: n/a; DD: 98%) API: silver sulfadiazine	Solution intercalation/ composite powder	The effective interaction between the organic and inorganic components The successful drug loading of clay/chitosan nanostructures	• XRD, high-resolution TEM and X-EDS analysis, FTIR, DSC/TGA, Zeta potential measurement, elemental analysis	[86]
MMT (CEC: 80.64 meq/100 g)/CS (Mw: n/a; DD: 98%) API: oxytetracycline hydrochloride	Solution intercalation and lyophilization/ composite powder	Good biocompatibility Enhancement of drug permeation	• FTIR, XRD, TGA, Zeta potential measurement, confocal laser scanning microscopy ♦ Cytotoxicity (MTT test), permeability studies (Caco-2 cells)	[110]
MMT (CEC: n/a)/CS (MW: 50–190 kDa; DD: 85%) API: chlorhexidine digluconate	Solution intercalation and lyophilization/ composite nanoparticles	Sustained release Mucoadhesivity Suitable for treatment of buccal infections	• FTIR, TGA, XRD, SEM ◊ In vitro drug release ♦ Antibacterial activity (the reference bacterial strain: *S. aureus*)	[90]
MMT (CEC: 120 meq/100 g)/CS (Mw: n/a; DD: n/a) API: chlorhexidine diacetate	Solvent casting/ composite films	Antimicrobial and antibiofilm activity Suitable for wound dressings	• XRD, TG, FTIR ♦ In vitro static biofilm assay (the reference bacterial strains: *S. aureus*, *S. epidermidis*, *P. aeruginosa*, *C. albicans*), cell viability assay ◊ Drug-loading determination, in vitro drug release	[104]
MMT (CEC: n/a)/CS (Mw: 90 kDa; DD: 81%) API: ofloxacin	Solution intercalation and ionic crosslinking with TPP/ nanocomposite beads	Improved drug loading and sustained drug release. The drug release rate of the beads was influenced by pH of the medium	• FTIR, XRD, SEM ◊ Entrapment efficiency, in vitro drug release (USP apparatus 2)	[105]
MMT (CEC: n/a)/N-quaternary CS (Mw: 100–300 kDa; DD: ≥ 90%) API: ciprofloxacin	Solution intercalation/ composite powder	Efficient drug encapsulation by N,N,N-triethyl CS composites Prolonged drug release and enhanced antibacterial activity	• FTIR, SEM, XRD, BET, DSC/TGA ♦ Antibacterial properties (the reference bacterial strain: S. *aureus*) ◊ Swelling studies, in vitro drug release study	[82]
BNT (CEC: n/a)/CS (MW: n/a; DD: 90%) ZnO (nanoparticles) Gelatin (prepared from *A. stellatus n. cyrenisis Berg* fish waste)	Solvent casting/ composite films	ZnO nanoparticles increased the porosity, hydrophilicity, and water absorption of the composite films High antibacterial activity Wound healing and epithelium regeneration	• FTIR, SEM ♦ Antibacterial activity (the reference bacterial strains: *S. aureus*, *P. aeruginosa*), evaluation of cell toxicity (proliferation of fibroblast cells, MTT assay), wound healing test (on rats), histology analysis ◊ Swelling degree, porosity analysis	[101]
Zinc (II)-MMT-organically modified (CEC: n/a)/CS (MW: n/a; DD: 95%) API: tea tree oil	Solution intercalation and adsorption saturation/ composite powder	Good loading capacity and sustained release for tea tree oil, alongside with good antibacterial effect against *E. coli*	• FTIR, SEM, XRD, N_2_ adsorption, Zeta potential measurement, X-ray photoelectron spectroscopy (XPS), DSC/TGA ♦ Antibacterial properties (the reference bacterial strain: *E. coli*) ◊ In vitro release study	[96]
HAL/CS (MW: 1000 Da; DD: 75.4%) API:/	Solution intercalation and lyophilization/ composite powder	Biocompatibility in vitro towards normal human dermal fibroblasts in an in vitro wound healing test (improved re-epithelialization effect)	• FTIR, DSC, XRPD, energy-filtered transmission electron microscopy (EFTEM) and electron energy loss spectroscopy (EELS), ultra-high resolution transmission electron microscopy (UHRTEM) and analytical electron microscopy (AEM), Zeta potential measurement ♦ In vitro biocompatibility, in vivo wound healing efficacy in the rat model, histological analysis	[92]
HAL/CS (MW: medium; DD: n/a) API: tetracycline, tetracycline hydrochloride	Solution blending/thermoresponsive hydrogel containing halloysite–chitosan composite	Sustained drug release and microbiological activity over a 6-week period	● Rheological testing ◊ In vivo investigation of efficacy (using a wound pocket creation model in dogs) • Drug loading and in vitro release, stability	[106]
HAL/CS (Mw: low, medium and high, according to the supplier’s data; DD: >75%) API: tetracycline hydrochloride	Solvent casting and evaporation/ nanocomposite films	Improved thermal stability and mechanical properties in comparison with corresponding CS films Sustained release of tetracycline hydrochloride during 8 h	• Determination of CS molar mass, rheological measurements of CS solutions, XRD, FTIR, DSC/TGA, SEM ◊ Determination of drug content, in vitro release study, Mathematical modeling of the release profiles, thickness measurement, mechanical properties (tensile strength (σ), elongation at break (ε), elastic modulus (E)), swelling study	[107]
HAL/CS (Mw: low; DD: n/a) API: gentamicin sulfate	Solution blending; hydrogels were formed by crosslinking the mixture solution with 10% TPP/ hydrogel composite	Sustained drug release and efficacy in reducing bacterial growth The addition of HAL improved mechanical properties of hydrogel nanocomposites	• SEM ◊ Bacterial inhibition growth test (the reference bacterial strains: *S. aureus*, *E. coli*), cytotoxicity assay ♦ Degradation analysis, tensile properties, swelling ratio, drug release study	[102]

**Table 2:** Most commonly used antibiotics for the treatment of topical/surface problems associated with potential or existing infections [115,116,117].

Antibiotic	Origin	Sensitive Microorganisms	Application for Topical Treatment	Application in Chitosan–Clay Nanocomposites
Clindamycin	A semisynthetic derivative of lincomycin	*Cutibacterium acnes* (*Propionibacterium acnes*)	Erythrasma, folliculitis, Fox–Fordyce disease, periorificial facial dermatitis, and rosacea	/
Fusidic acid	A steroid-like structure derived from the fungus *Fusidium coccineum*	Gram-positive bacteria such as *Staphylococcus* sp. and *Corynebacterium* sp.	Impetigo, erythrasma, and pitted keratolysis	/
Gentamicin	An aminoglycoside antibiotic; derived from *Micromonospora purpurea*	Mostly Gram-negative like *Pseudomonas*, *Proteus*, and *Escherichia coli*, and Gram-positive like *S. aureus*	Impetigo and folliculitis	[102]
Metronidazole	Synthetic nitroimidazole	Most anaerobic bacteria and protozoa	Rosacea, benign and malignant ulcers, including pressure sores infected with anaerobes	/
Mupirocin	Derived from *Pseudomonas fluorescens*; unique structure, distinct from any other antibiotics	Ineffective against most aerobic Gram-negative bacteria and anaerobes, but active against penicillinase-producing and methicillin-resistant strains of *S. aureus*	Impetigo, secondarily infected eczema, infected wounds with strains of *S. aureus* or *Streptococcus pyogenes*; nasal colonization of methicillin-resistant *S. aureus* (MRSA)	/
Nadifloxacin	A synthetic quinolone	Broad-spectrum bactericidal activity (aerobic Gram-positive, Gram-negative, and anaerobic bacteria, including *P. acnes* and *S. epidermidis*)	Active against MRSA, high potential as an alternative for topical antibiotic treatment in bacterial skin infection	/
Neomycin	A bactericidal aminoglycoside antibiotic, which acts by inhibiting bacterial protein synthesis	Mostly Gram-negative organisms like *Proteus*, *E. coli*, *Serratia*, and *H. influenzae*	Superficial pyodermas, minor wounds, and secondarily infected dermatitis	/
Retapamulin	A semisynthetic antibiotic	Isolates resistant-to-therapy beta-lactams, macrolides, quinolones, topical fusidic acid, and mupirocin	Impetigo, the short-term treatment of impetigo and infected small lacerations, abrasions, and sutured wounds	/
Silver sulfadiazine	A topical sulfonamide	A wide spectrum of activity against both Gram-positive and Gram-negative bacteria	Prevention and treatment of wounds caused by serious burns	[86,111]
Tetracycline	Derived by *Streptomyces genus* of *Actinobacteria*	Exhibits various bacteriostatic effects against many aerobic and anaerobic bacterial genera, both Gram-positive and Gram-negative	Treatment of several infections, including acne and rosacea	[106,107]
